# Diagnosis of Phantom Abdominal Tumors

**Published:** 1872-02

**Authors:** 


Diagnosis of Phantom Abdominal Tumors.—In an interesting clinical lecture published in the Medical Times, Dr. J. M. De Caster states that the most efficient way of diagnosticating phantom tumors of the abdomen is by the use of an anæsthetic. When under the influence of ether, a phantom tumor must disappear, and thus remove all doubt as to the nature of the case. He says, "Not in a single instance have I found these apparent tumors remain when the patient is under the influence of ether or chloroform, though they re-appear, and quickly, when he passes from under its influence." Thus, we can control the diagnosis not only of phantom tumors, but muscular contractions of the abdomen, not unfrequently feigned, and which high authority declares a source of difficulty almost impossible to get over.

Hebra, the celebrated Professor of Dermatology at Vienna, in the course of his clinical lectures (*Gazette Médicale*), made the following curious statements: "1. About ninety-six per cent. of children who have been affected with prurigo die of phthisis. 2. Women who have been for a long time sufferers from eczema of the scalp become, in old age, the subjects of cancer. 3. Variola and varicella are essentially the same disease. He has seen a severe epidemic of variola developed from a single case of varicella."

